# Comparative efficacy of hospital disinfectants against nosocomial infection pathogens

**DOI:** 10.1186/s13756-020-00781-y

**Published:** 2020-07-22

**Authors:** Fahim Amini Tapouk, Ramin Nabizadeh, Nezam Mirzaei, Nima Hosseini Jazani, Mahmood Yousefi, Mohamad Amin Valizade Hasanloei

**Affiliations:** 1 Department of Environmental Health Engineering, School of Public Health, International Campus, Tehran University of Medical Sciences (IC-TUMS), Tehran, Iran; 2 Department of Environmental Health Engineering, School of Public Health, Tehran University of Medical Sciences, Tehran, Iran; 3 Center for Air Pollution Research (CAPR), Institute for Environmental Research (IER), Tehran University of Medical Sciences, Tehran, Iran; 4 Department of Environmental Health Engineering, Faculty of Health, Kashan University of Medical Sciences, Kashan, Iran; 5 Department of Microbiology, Faculty of Medicine, Urmia University of Medical Sciences, Urmia, Iran; 6 Department of Environmental Health Engineering, School of Public Health, Iran University of Medical Sciences, Tehran, Iran; 7 Clinical Research Development Unit of Emam Khomeini Hospital, Urima University of Medical Sciences, Urmia, Iran

**Keywords:** Health care associated infections, nosocomial infection pathogens, High level disinfectants, Minimum inhibitory concentration (MIC), *Enterococcus faecalis* ATCC 29212, *Burkholderia cepacia* ATCC 10673

## Abstract

**Background:**

Due to the increasing rate of hospital-acquired infections, it is essential to select appropriate disinfectant agents. In this study, the efficacy of hospital disinfectants against nosocomial infection pathogens was compared.

**Methods:**

High level disinfectants (Steranios 2%, Deconex HLDPA, and Microzed Quatenol) were tested for their antibacterial effects by determining their minimum inhibitory (MIC) and minimum bactericidal concentrations (MBC) against *Enterococcus faecalis* ATCC 29212 and *Burkholderia cepacia* ATCC 10673.

**Results:**

*E. faecalis*, as gram-positive bacterium, was more susceptible to high level disinfectants compared to gram-negative *B.cepacia.* The MIC = MBC values of 2% Steranios, Deconex HLDPA and Microzed Quatenol against *E. faecalis* and *B.cepacia* were 0.31, 9.77, 2.2 mg/L and 9.8, 78.13, 70.31 mg/L, respectively.

**Conclusions:**

According to the findings of this study, the most effective disinfectants against both *E. faecalis* and *B.cepacia* were Steranios 2%, Microzed Quatenol, and Deconex HLDPA in order. Considering the importance of these bacterial strains in healthcare-associated infections, the use of these effective disinfectants is recommended in the hospitals.

## Introduction

Healthcare-associated infections with approximately 7.1 million annual cases and 99,000 annual deaths in the United States have been recognized as a group of important causes of morbidity and mortality in admitted patients [1]. The patient’s microbiota is believed to be the major source of infections that are transmitted from the medical staff and the hospital environment. Approximately 20–40% of these infections are transmitted via hands [2, 3]. Hospital-acquired infections are a major concern in health care centers, which may also increase the length of hospitalization, costs and mortality rate in patients [4]. Contamination of the hospital environment plays a significant role in the transmission of several pathogens such as Methicillin-resistant *Staphylococcus aureus* (MRSA) and Vancomycin-resistant Enterococcus (VRE) [4, 5]. Another bacterial species that has been important in recent years as a multi-drug resistance agent of hospital infections is *Burkholderia cepacia*, which was first described in patients with systemic fibrosis in the late 1970s. *Burkholderia cepacia* isolates are usually resistant to various classes of antibiotics and disinfectants [6]. Enterococci have been long recognized as important human pathogens and *Enterococcus faecalis* is one of the most common and life-threatening causes of nosocomial infections [7, 8]. In the healthcare setting *Enterococcus faecalis* was known as an important cause of hospital-acquired bacteremia and mortality among the elderly and patients with major disorder conditions [9].

To prevent infections, it is important to remove bacterial biofilms from contaminated surfaces equipment and the medical environment [10]. Contaminated hospital surfaces can transmit the pathogens to the patients, so cleaning and disinfecting surfaces will break this chain [1]. Decontamination methods such as sterilization (physical or chemical) and disinfection processes are key principles of successful programs in the control of nosocomial infections [6]. Persistence of hospital infections may be in part due to the inappropriate and inadequate use of decontamination methods [11]. In hospitals, medical instruments are classified into three categories of critical, semi-critical, and non-critical based on the risk of infection transmission and contact with the patient’s body. Critical items enter sterile tissues so the use of these devices increases the risk of infection if they are not properly disinfected; therefore, in these cases applying liquid chemical disinfectants is essential after reuse [7, 12]. The main purpose of using disinfectants in hospitals is to reduce the risk of sporadic and epidemic infections. To achieve this goal, different methods and various disinfectants are recommended [13, 14]. Furthermore, due to the changing patterns of resistance in nosocomial pathogens, daily revision of the efficacy of disinfectants and chemical sterilizers against multi-drug resistant pathogens seems necessary [15].

However, due to the accessibility of various and different brands of disinfectant products, selection of the right one has become a challenge for environmental health experts and infection control supervisors in the health-care settings. The choice of disinfectants according to the manufacturer’s instructions is not only unreliable but also causes irreversible damages to instruments and increases the rate of outbreaks related to the disinfectant-resistant pathogens in hospitals in some cases. As an environmental health expert working in a hospital, i have an unpleasant experience in this field because one of our endoscopic devices was seriously damaged after a few months of using the peroxyacetic acid (PAA) solution.

Therefore, it is necessary to investigate the sensitivity of bacterial pathogens to currently used disinfectants by different methods such as determining the minimum inhibitory concentration (MIC) and minimum bactericidal concentration (MBC). The aim of the present study was to determine the efficacy of high level disinfectants against two multidrug resistant bacterial pathogens, *E. faecalis* (ATCC 29212) and *B. cepacia* (ATCC 10673), by determining the MIC and MBC of each disinfectant and comparing their efficacy with on another.

## Methods

### Materials

This experimental study was conducted in the Diagnostic Microbiology Lab of Imam Khomeini Hospital, Urmia, Iran in 2017. Mueller-Hinton agar (MHA), Mueller-Hinton broth (MHB), and skim milk containing 15% sterile glycerol (all purchased from Merck, Germany) were used for cultivation the bacteria, determining the MICs and MBCs, and storing the isolates. *B. cepacia* ATCC 10673 and *E. faecalis* ATCC 29212 were obtained from Iranian Biological Resource Center (IBRC) and Persian Type Culture Collection (PTCC) respectively and stored in skim milk containing 15% glycerol at − 20 °C. Three disinfectants including Deconex HLDPA (DH), Steranios 2%, and Microzed Quatenol (MQ) were obtained from Armanbehboud, Ayriaborna, and Pharmedparto Company, respectively. These disinfectants are applied routinely as liquid chemical sterility agents in operation rooms and bronchoscopy and endoscopy units in our hospital. The basic compounds of each disinfectant are shown in Table 1.

**Table 1:** Basic compounds of tested antimicrobial agents

Brand of biocidal agents	DH	2% Steranios	MQ
**Basic compounds biocidal agents**	1200 PPM of Peracetic acid with Surfactant Compounds	2% v/v glutaraldehyde solution	Alcohol based with three types of quaternary ammonium compounds

### Preparation of serial dilutions of disinfectants

**Fig. 1:**
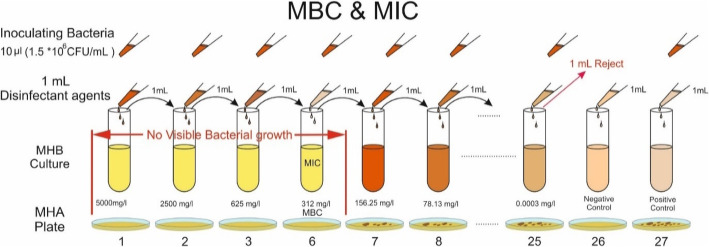
Broth Dilution Method for determining MIC and MBC values of disinfectants against tested bacteria. MIC is equal to MBC = 312 mg/L. The Mueller-Hinton broth (MHA) and Mueller-Hinton agar were used for determining MIC and MBC, respectively. The inoculum size was 1.5 × 10^6^ CFU/mL

Briefly, serial two-fold dilutions of disinfectants were prepared in sterile MHB. To prepare various concentrations of disinfectants***,*** 27 sterile test tubes were numbered from 1 to 27 (As shown in Fig. 1). One mL of sterile MHB was added to each tube in aseptic conditions. One mL of testing disinfectant (undiluted) was poured into the tube No. 1; after mixing, serial two-fold dilutions were prepared by adding 1 mL of the contents of each tube to the next one. The process continued to the tube 25 (except for tubes 26 and 27). From tube 25, one milliliter was poured out, so the volume of all tubes was adjusted to 1 mL. Tubes 26 and 27 were considered as negative (disinfectant + MHB) and positive (1.5 × 10^6^ CFU of bacterial inoculums + MHB) controls, respectively. This procedure was done for all disinfectants. All experiments were done in triplicates for each tested disinfectant and each bacterial strain.

### Preparation of the McFarland turbidity standards

McFarland turbidity standards were prepared as references for estimating the amount of bacterial inoculum. In this study, 0.5 McFarland turbidity standard was prepared [16] by adding 0.5 mL of 0.048 M barium chloride (1.17% w/v BaC_l2_. 2H_2_O) solution to 99.5 mL of 0.18 H_2_SO_4_ (1% v/v) and mixing with a magnetic stirrer. To compare the 0.5 McFarland turbidity standards with bacterial suspension, about 10 mL of this standard was transferred to a glass capped tube. The prepared solution should not be frozen and can be kept for up to 6 months. Vertex should always be applied before the use of turbidity standards and the standard cannot be used if rust or color change is observed or after the expiration date.

### Bacteria suspension

The 0.5 McFarland standard [17] was used as a reference to compare the turbidity of bacterial inoculum in order to adjust the approximate cell density. Fresh cultures of bacterial strains were prepared on the MHA medium by overnight incubation at 37 °C. Then, 3–4 colonies of each bacterial strain were picked up with a sterile cotton swab and transferred to 4–5 ml of sterile buffered saline. After mixing perfectly, turbidity was visually compared with the 0.5 McFarland standard. After adjustment, the bacterial amount in the broth culture was approximately 1.5 × 10^8^ colony-forming units per mL (CFU/mL).

### Determination of MIC and MBC

The susceptibility of the bacterial strains was determined using the modified microdilution method recommended by NCCLS (National Committee for Clinical Laboratory Standards) guidelines [17]. MIC_24_ is defined as the lowest concentration of a biocide agent which has an inhibitory effect on the cultivated bacterium, causing no turbidity in the test tube after incubation at 37 °C for 24 h. After preparing serial dilutions, 10 μl (1.5 × 10^6^ CFU/mL) of 0.5 McFarland adjusted fresh bacterial culture was inoculated to each dilution and tubes were incubated at 37 °C for 24 h. Then, the test tubes were examined for their turbidity. The lowest concentration of a disinfectant that inhibited the bacterial visible growth was considered as MIC. To determine the minimum bactericidal concentration of disinfectants, 5 μl of each tube was sub-cultured on the surface of MHA at 37 °C for 24 h and then colony count was done. MBC_24_ was considered as the concentration of the disinfectant that prevented the growth of 99.9% (3-log) of the bacteria as growing less than 15 colonies on MHA.

## Results

The values of MIC_24_ & MBC_24_ for the tested disinfectants were as follows:

### Steranios 2%

In our hospital Steranios 2% containing 2% glutaraldehyde is commonly used as a disinfectant or sterilizer for critical and semi-critical items in an exposure time and concentration dependent manner. It is available in both forms of liquid or gas, but the liquid form is used in our hospital. The MIC and the MBC of Steranios 2% for *E. faecalis* ATCC 29212 was 0.31 mg/L. At sub-MIC doses with a lower concentration of the biocide, the tubes were turbid and the bacteria grew easily after cultivating on solid media. The MIC and the MBC of Steranios 2% for *B. cepacia* ATCC 10673 was 9.83 mg/L (Table 2).

**Table 2:** MIC_24_ and MBC_24_ values of investigated disinfectants for *B. cepacia* ATCC 10673 and *E. faecalis* ATCC 29212

MIC_**24**_ and MBC_**24**_ (mg/L)			
Bacterial strains	2%Steranios	DH	MQ
	MIC_**24**_ = MBC_**24**_	MIC_**24**_ = MBC_**24**_	MIC_**24**_ = MBC_**24**_
***B. cepacia ATCC 10673***	9.83	78.13	70.31
***E.faecalis ATCC 29212***	0.31	9.77	2.2

### Deconex HLDPA (DH)

DH is applied as a high-level disinfectant to sterilize reusable medical devices such as flexible endoscopes in operating rooms and intensive care units. As shown in Table 2, The MIC and the MBC of DH for *E. faecalis* ATCC 29212 and *B. cepacia* ATCC 10673 was 9.77 mg/L and 78.13 mg/L, respectively.

### Microzed Quatenol (MQ)

**Fig. 2:**
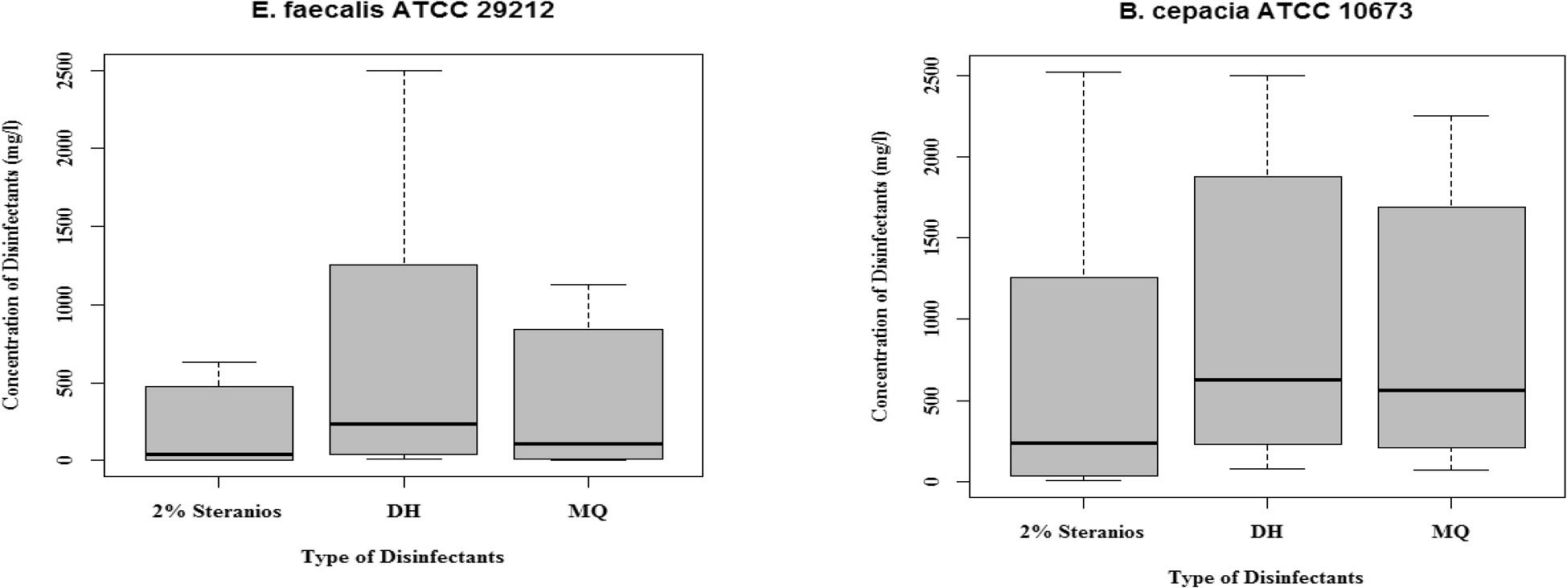
Medians of tested disinfectants (Steranios 2%, DH and MQ) concentrations as MIC = MBC (mg/L) on *B. cepacia* ATCC 10673 and *E. faecalis* ATCC 29212

MQ antimicrobial agents are ready-to-use disinfectants in hospitals, clinics, and dental settings that are applied in the form of spray on sensitive surfaces of electronic equipment, cameras, work tables and dental units in health-care centers. As shown in Table 1, in order to increase the efficacy, MQ contains alcohol and three types of quaternary ammonium compounds. The MIC and the MBC values of MQ for *E. faecalis* ATCC 29212 and *B. cepacia* ATCC 10673 were 2.2 mg/L and 70.31 mg/L, respectively. The MBC values for both strains were equal to MIC values. According to Table 3, the concentration ranges for Steranios 2%, DH, and MQ were 5035–0.0003, 5000–0.0003, and 4500.0–0.0003 mg/L, respectively. Figure 2 presents the median values of data for disinfectants.

**Table 3:** Concentration ranges and breakpoints of disinfectants used to determine the MIC_24_ and MBC_24_ values for *B. cepacia* ATCC 10673 and *E. faecalis* ATCC 29212

Tube NO	MIC_**24**_ = MBC_**24**_ (mg/L)			MIC_**24**_ = MBC_**24**_ (mg/L)		
	***E. faecalis*** ATCC 29212			***B. cepacia*** ATCC 10673		
	2% Steranios	DH	MQ	2% Steranios	DH	MQ
**1**	5035.00	5000.00	4500.00	5035.00	5000.00	4500.00
**2**	2517.50	2500.00	2250.00	2517.50	2500.00	2250.00
**3**	1258.75	1250.00	1125.00	1258.75	1250.00	1125.00
**4**	629.38	625.00	562.50	629.38	625.00	562.50
**5**	314.69	312.50	281.25	314.69	312.50	281.25
**6**	157.34	156.25	140.63	157.34	156.25	140.63
**7**	78.67	78.13	70.31	78.67	MIC = MBC = 78.13	MIC = MBC = 70.31
**8**	39.34	39.06	35.16	39.34	39.06 TO*	35.16 TO*
**9**	19.67	19.53	17.58	19.67	19.53	17.58
**10**	9.83	MIC = MBC = 9.77	8.79	MIC = MBC = 9.83	9.77	8.79
**11**	4.92	4.88 TO*	4.39	4.92 TO*	4.88	4.39
**12**	2.46	2.44	MIC = MBC = 2.20	2.46	2.44	2.20
**13**	1.23	1.22	1.10 TO*	1.23	1.22	1.10
**14**	0.61	0.61	0.55	0.61	0.61	0.55
**15**	MIC = MBC = 0.31	0.31	0.27	0.31	0.31	0.27
**16**	0.15 TO*	0.15	0.14	0.15	0.15	0.14
**17**	0.08	0.08	0.07	0.08	0.08	0.07
**18**	0.04	0.04	0.03	0.04	0.04	0.03
**19**	0.02	0.02	0.02	0.02	0.02	0.02
**20**	0.0096	0.0095	0.0086	0.0096	0.0095	0.0086
**12**	0.0048	0.0048	0.0043	0.0048	0.0048	0.0043
**22**	0.0024	0.0024	0.0021	0.0024	0.0024	0.0021
**23**	0.0012	0.0012	0.0011	0.0012	0.0012	0.0011
**24**	0.0006	0.0006	0.0005	0.0006	0.0006	0.0005
**25**	0.0003	0.0003	0.0003	0.0003	0.0003	0.0003
**26**	Negative controls (disinfectant + MHB)					
**27**	Positive (inoculum + MHB)					

*TO Turbidity was observed

*At this concentration and lower (Up to tube number 25) turbidity was observed and indicates a non-bactericidal range of disinfectants

## Discussion

Increased resistance of microorganisms to antibiotics and disinfectants and emergence of biocide-resistant bacterial strains have made it difficult to eliminate them. Disinfectants are effective in reducing health care associated infections, but the correct choice of disinfectants considering the wide range of efficacy against hospital pathogens is important and essential in this process [15, 18]. The purpose of this study was to evaluate the efficacy of three hospital disinfectants in two strains of pathogenic bacteria based on MIC and MBC tests. According to Tables 2, 3, and Fig. 2 the disinfectants did not have an equal efficacy in the tested strains and *B. cepacia* showed more resistance to all tested disinfectants compared to *E.faecalis*. It can be concluded that the same disinfectants should not be used for all wards, sections and places in healthcare settings. The results of this study showed that under the same conditions, different disinfectants revealed various efficacies and diverse ranges of activity against different bacterial strains. On the other hand, equal concentrations of disinfectants in the same application situation may have different effects on different types of bacteria. Therefore, before choosing and applying any disinfection in hospitals, it is absolutely necessary to measure the MIC and MBC values of daily used disinfectants against common life-threatening bacterial agents or healthcare associated infections.

MIC and MBC assay tests are tools to determine the effectiveness of disinfectants against micro-organisms and to determine the sensitivity of bacteria in health care settings [19, 20]. If MIC and MBC values of a disinfectant are small enough, bacterial growth is inhibited at low concentrations of the disinfectant; in this case, the disinfectant is stronger, more effective, and suitable. In this situation, if the disinfectatnt is inexpensive (reasonable price), it is considered the best one compared to others. MIC values are usually less than MBCs, because higher concentrations of an antibacterial agent are needed for bactericidal versus bacteriostatic effects in most cases. MIC is the lowest concertation that inhibits the growth of tested bacteria, whereas MBC is usually a relatively higher concentration of the disinfectant that kills the microorganism. According to our findings, MIC and MBC of the two disinfectants were equal values in all experiments; this could be due to the optimal antibacterial properties of these biocidal agents. The MIC and MBC results of MQ showed that in the same conditions, it has different antibacterial effects on tested strains. MQ, at a concentration of MIC = MBC = 70.31 mg/L, reduced the growth of *B. cepacia* ATCC 10673 to 3-Log (99.9%), whereas a 3-Log reduction (99.9%) in growth occurred at MIC = MBC = 2.2 mg/L for *E. faecalis* ATCC 29212. Therefore, it can be concluded that either *E. faecalis* ATCC 29212 is more susceptible than *B. cepacia* ATCC 10673 to this disinfectant or the antibacterial effect of MQ is weaker on the *B. cepacia* ATCC 10673. Lower sensitivity of gram-negative versus gram-positive bacteria is mostly attributed to differences in chemical composition of gram-negative and gram positive cell walls, resulting in different bacteria behaviors against disinfectants. The cell wall of the gram-negative bacteria contains an outer membrane [21] that partly prevents the penetration of disinfectants and antibiotics into the bacterium. Acquisition of certain types of plasmids can make gram negative bacteria more resistant to biocides because of the production of detoxifying enzymes [22]. In this study, *B. cepacia* ATCC 10673 (gram negative) was more resistant to disinfectants compared to *E. faecalis* ATCC 29212 (gram-positive), but the mechanism of resistance was not investigated. Therfore, more detailed studies are required to explore the mechanism of resistance of the studied bacteria to these disinfectants.

*E.faecalis* showed a high sensitivity to Steranios 2% (MIC = MBC = 0.31 mg/L). However, in the same conditions, the lowest bacteriostatic and bactericidal concentration of Steranios 2% for *B. cepacia* was 9.83 mg/L. It was also observed that *E.faecalis* was less resistant to lower concentrations of disinfectant. So, in hospital wards where *E.faecalis* is prevalent, even low concentrations of Steranios 2% can be applied for eradication of this strain. This recommendation should be taken with cautionbecause the rate of resistance to Steranios 2% in other prevalent hospital-acquired pathogens should be determined and considered as well. The major components of Steranios 2% are glutaraldehydes, which are used as high-level disinfectants in healthcare settings for sterilizing medical instruments such as endoscopes, lenses, rubber, plastic, etc. Glutaraldehyde has no corrosive properties and its shelf- life is 14 days [1]. It stimulates the skin, eyes, and respiratory tract and there are occasional health problems such as occupational asthma in dealing with glutaraldehyde [23]. Therefore, when glutaraldehyde is used as a disinfectant, care should be taken and attention should be paid to ventilation. The range of bactericidal (MIC) or bacteriostatic concentrations (MIC) of Steranios 2% was 5035–9.83 mg/L for *B. cepacia* and 5035–0.31 mg/L for *E.faecalis*. Pariscila et al. [11], showed that the MIC values of glutaraldehyde for *E. cloacae*, *B. subtilis* and *E. coli* were in the range of 2750–3750 mg/L. Whereas, our results show that the MIC values were markedly lower in the present study (0.31 mg/L vs. 2750–3750 mg/L).

Another disinfectant in this study was DH whose effectiveness was compared with other tested disinfectants. Its main ingredient is peracetic acid (PAA). Peracetic acid is a suitable alternative for decontamination of heat-sensitive medical equipment. This antibacterial agent has a rapid activity against bacterial spores, but it has corrosive effects on some metal equipment [24]. The present study also showed (Table 3) that *B. cepacia* was generally less sensitive to DH than *E. faecalis*. At 19.53 mg/L, DH showed marked antibacterial effects against *E. faecalis* whereas *B. cepacia* was more tolerant to it. The bactericidal concentration range of DH was 5000–156.25 mg/L for *B. cepacia* and 5000–19.53 mg/L for *E. faecalis*. So, it can be concluded that 19.53 mg/L of DH has bactericidal effects on 99.9% of *E. faecalis* population, but the same concentration is ineffective on *B. cepacia*. In another study, the MIC range of peracetic acid for *B. cepacia* and some gram-negative strains was 2310–4491 mg/L [19] which was in the scope of our study. The median concentration of Sterniose 2% against *E. faecalis* and *B. cepacia* was lower than DH and MQ, respectively (Fig. 2). In addition *E. faecalis* needs a lower bactericidal concentration range of disinfectants compared to *B. cepacia* and a lower median of a disinfectant is the most effective against pathogens.

MIC and MBC represent the minimum inhibitory and minimum bactericidal concentration of disinfectants at 24 h [25], However, to minimize the risk of infection in hospital environments, there is a need for a potent antibacterial activity in very shorter exposure time for disinfection of critical equipment. Therefore, future studies should be conducted to determine the bacteriostatic and bactericidal effects of disinfectants at short time exposures, including 5, 10, and 20 min. Due to the limited number of critical items (e.g. endoscopic instruments, bronchoscopes, arthroscopy devices, etc.) in our hospital and the physicians’ interest in visiting more patients in less time, it is recommended to determine the effective concentrations of various disinfectants (low, intermediate, and high level) in short contact times with pathogenic microorganisms. In this study, the bacteriostatic and bactericidal effects of three different disinfectants were investigated on reference isolates of *E. faecalis* and *B. cepacia.* It is obvious that bacterial isolates from patients, hospital personnel, or hospital environment are usually more resistant to antimicrobial agents, so further studies in a larger number of hospital isolates are recommended. The present study showed the different efficacy of three classes of disinfectants on two different strains of bacteria. Considering the variable effects of disinfectants on several nosocomial pathogens, alternate use of disinfectants in hospital wards is highly recommended. These findings could be used in hospitals or health care settings where the efficacy of disinfectants is important.

## Conclusion

In conclusion the most effective surface and instrument disinfectants against both *E. faecalis* and *B. cepacia* were Steranios 2% (MIC = 0.31 mg/L) > MQ (MIC = 2.20 mg/L) > DH (MIC = 9.77 mg/L) and Steranios 2% (MIC = 9.83 mg/L) > MQ (MIC = 70.31 mg/L) > DH (MIC = 78.13 mg/L), respectively. The use of non-lethal doses (sub-MIC) of disinfectants not only does not stop the growth of these pathogens but also increases the resistance level of microorganisms to disinfectants. The use of higher concentrations of disinfectants (above MBC) only results in overconsumption of disinfectants and is not cost-effective.

## Data Availability

The datasets generated during and/or analyzed during the current study are not publicly available due to joint research and development with the company but are available from the corresponding author on reasonable request.
